# Effect of Mindfulness-Based Group Counseling on Depression in Infertile Women: Randomized Clinical Trial Study

**DOI:** 10.22074/ijfs.2020.5785

**Published:** 2020-02-25

**Authors:** Fatemeh Kalhori, Seyedeh Zahra Masoumi, Farshid Shamsaei, Younes Mohammadi, Mahnaz Yavangi

**Affiliations:** 1 Consultation in Midwifery, Department of Midwifery, Hamadan University of Medical Sciences, Hamadan, Iran; 2 Mother and Child Care Research Center, School of Nursing and Midwifery, Hamadan University of Medical Sciences, Hamadan, Iran; 3 Mother and Child Care Research Center, Hamadan University of Medical Sciences, Hamadan, Iran; 4 Modeling of Noncommunicable Diseases Research Center, Department of Epidemiology, School of Public Health, Hamadan University of Medical Sciences, Hamadan, Iran; 5 Endometrium and Endometriosis Research Center, Hamadan University of Medical Sciences, Hamadan, Iran

**Keywords:** Counseling, Depression, Female, Infertility, Mindfulness

## Abstract

**Background:**

Assisted reproductive technologies (ARTs) such as *in vitro* fertilization (IVF) can lead to depressive symptoms in infertile women due to their low success and high costs. Mindfulness-based group counseling can decrease depressive symptoms by increasing mental concentration. The aim of the present study was to evaluate the effect of mindfulness-based group counseling on depression in infertile women undergoing IVF.

**Materials and Methods:**

The present clinical trial included 90 infertile women undergoing IVF treatment in an infertility center in 2016. Women were divided into two groups, intervention and control. Both groups completed a demographic questionnaire and the Beck depression inventory (BDI). Eight 90-minute sessions (two each week) of mindfulness-based group counseling were held with the intervention group, while the control group received treatment as normal. Following the intervention, the BDI was again completed by both groups. The data were analyzed and independent t tests and, paired t tests conducted at a significance level of P<0.05.

**Results:**

No statistically significant demographic differences were observed between the two groups. Women in the control group had a somewhat lower depressive symptom score than the intervention group before the intervention. However, compared with before, the depressive symptom score among women in the intervention group decreased significantly (48%) (P<0.001) after the intervention. In contrast, the depressive symptom score in control women was higher after the intervention than before.

**Conclusion:**

According to the findings of the present research, mindfulness-based group counseling is able to reduce depressive symptoms in infertile women under IVF treatment. Therefore, group counseling sessions are suggested for all depressed women undergoing infertility treatment (Registration number: IRCT2015082013405N14).

## Introduction

Primary infertility is defined as an inability to conceive after 1 year of unprotected sex (without using contraceptives), and can be related to the male or female partner or both (1). Worldwide, more than 80 million people are infertile (2).

The WHO states that inability to bear a child, either due to the inability to become pregnant or the inability to carry a pregnancy to a live birth following either a previous pregnancy or a previous ability to carry a pregnancy to a live birth. In 2010, among women 20-44 Y of age who were exposed to the risk of pregnancy, 1.9% (95% uncertainty interval 1.7%, 2.2%) were unable to attain a live birth (primary infertility). Out of women who had had at least one live birth and were exposed to the risk of pregnancy, 10.5% (9.5%, 11.7%) were unable to have another child (secondary infertility) (3).

Prevalence varies between countries with a global average of 12 to 15%. Infertility can be divided into two groups; primary (no conception occurring over the past year) and secondary infertility (conception without giving birth to a living child). In Iran the prevalence of primary infertility based on the WHO's clinical, epidemiological and demographic definitions. is 20.2, 12.8 and 9.2%, respectively (2, 3). At a global level, the primary infertility rate is 0.6 to 3.4%, and the secondary infertility rate is 8.7 to 32.6%. In Iran, the mean primary and secondary infertility rates are 10.6% and 2.7%, respectively (4).

In response to the infertility rate, rapid progress in reproductive medicine has contributed to new technologies associated with the care and treatment of infertile couples across the world (4). Assisted reproductive technology (ART), including a wide range of treatments and approaches, is a common and successful treatment in many countries (5). One of the techniques is *in vitro* fertilization (IVF), a complex series of procedures commencing with extreme and controlled ovarian stimulation by exogenous gonadotropin, including techniques wherein fertilization is undertaken using intra-cytoplasmic injection of sperm, gamete transference to the fallopian tube, transfer of zygote into the fallopian tube, and the transfer of the peritoneal tube by laparoscopy (6). Epidemiological findings have documented high levels of depression in different countries. In 1990, the prevalence of depression was 472 million worldwide with, around 5 million in Iran, showing the high prevalence and importance of depression disorder on both global and national scales (7). Depression can increase during periods of infertility, and it is estimated that approximately 86% of infertile couple experience depression (8). One study showed that although the events and conditions that reveal depression, anxiety and stress differ from person to person, depression in infertile women is twice that in fertile women (9).

Although most people who seek infertility treatment seem to be emotionally stable, infertility is known to be a life-long crisis. Most infertile people have to deal with depression, feelings of loss and guilt, detachment, meaninglessness, and sexual and marriage problems. In addition, physical, psychological and economic problems associated with ART influence the psychological stability of couples (10). Psychological treatments administered along side infertility treatment programs, make infertile women more resistant to stress, increase the effectiveness of infertility treatments, and encourage infertile patients to follow the treatment by enhancing their mental health (11).

Studies conducted in infertile women have indicated the positive effect of counseling and psychological interventions on improving life quality (12). Mindfulness-based interventions are a common type of cognitive-behavioral therapy. Mindfulness is a form of meditation rooted in the eastern religious rituals, especially those related to Buddhism (13). Mindfulness is one of which is high awareness, focusing on the reality of the present, accepting and acknowledging it, regardless of the thoughts about the situation or emotional reactions to the situation (14). In essence, mindfulness consists of an informed and nonjudgmental sense of what is happening now (15). Pots et al. (16) document the important role of the learned skills of attention control in mindfulness meditation in preventing depression relapse. Based on their information processing theory, those who have experienced major periods of depression are susceptible to relapse when faced with a dysphoric state, because these states can activate the depressed thinking patterns of the period of depression. In this study, Mindfulness-Based Cognitive Therapy (MBCT) was employed as it includes meditation techniques for mindfulness and meditation along with daily activities for depression (17).

Given the problems of infertile women, such as depression, the prevalence of infertility and the few studies conducted in Iran, especially on the impact of group counseling on infertility and the lack of comprehensive therapeutic methods in the field of counseling, the present study aimed to evaluate the effect of mindfulness-based group counseling on depression in infertile women under IVF treatment.

## Materials and Methods

### Instruments

#### Demographic questionnaire

Demographic characteristics were assessed using a questionnaire designed by the researchers. It included questions about the personal characteristics of infertile women and their partners (10 questions), expenditures and the existence of health insurance coverage (2 questions), duration of marriage, duration of infertility, number of infertility years, frequency of IVF use and questions regarding psychiatric history (5 questions). Personal information included: first and last name, place of residence, age, employment, and education of the women and their partners, and monthly family income. Infertility was either primary (no pregnancy) or secondary (only pregnant once). Questions related to psychiatric histories included history of admission to psychiatric hospitals, history of mental illness, and use of psychiatric drugs and narcotics.

#### Beck depression inventory

The second Beck depression inventory (BDI-II) is a depression inventory and a self-report index for measuring depression symptoms in different clinical and nonclinical populations. Published in 1996 the second edition of BDI-II inventory was developed in response to the American Psychiatric Association’s publication of the Diagnostic and Statistical Manual of Mental Disorders, Fourth Edition (DSM-IV), which changed many of the diagnostic criteria for Major Depressive Disorder (American Psychiatric Association, 1994). This inventory is a 21-item self-reported measure of depression with 15 questions related to psychological symptoms and 6 questions related to physical symptoms. Time frame for BDI-II is consistent with the 1-2 weeks time frame for major depressive disorders in DSM-IV. All the questions assess the severity of the disorder based on a Likert scale (0-3). The total score of a participant is obtained by aggregating the scores of all questions of 0 to 63. Based on Beck's suggested scoring, a score of 0-9 indicates the absence of depression, 10-18 indicates mild to moderate depression, 19-29 moderate to severe depression, and 30-63 severe depression. Since the results of many studies of the BDI-II have shown its validity and reliability in different countries, the same questionnaire was used in the present research. Rajabi and Karjo (18) (according to Karmoudi study) obtained a Cronbach’s alpha coefficient of 0.91 for a student sample and reliability coefficients of 0.90, 0.87, and 0.44 for the whole questionnaire, the cognitive-emotional factor, and the physical factor, respectively. In the study of Khormaei et al. (19) (according to Dobson and Mohamadkhani study), the reliability coefficient measured as Cronbach's alpha was 0.91 and Goodarzi reported a Cronbach’s alpha of 0.84 for internal consistency. In this study, Cronbach’s alpha coefficient for the reliability of the BDI-II was 0.78.

### Procedures

The present clinical trial (IRCT2015082013405N14) which included a pre-test, post-test, and control group was conducted in women with diagnosed primary infertility who were in the early stages of IVF. Inclusion criteria were age 25-40 years, high school education or more, residency in Hamedan, no psychiatric hospital admissions, no addiction, no neurological or other progressive diseases, and no psychiatric drug use. Level of depression [mild mood disturbances, moderate depression, and severe depression (up to 63)] were determined using cut-off points of the BDI-II. Exclusion criteria were absence from more than two counseling sessions in the test group, natural pregnancy and no use of ART during treatment, and incidence of physical or psychological illness during the study. Women meeting the inclusion criteria and who agreed to participate in the study were selected prior to IVF treatment. Based on the eligibility criteria, a convenience sampling approach was used to select the participants. Among the 120 women who met the inclusion criteria, 90 women were enrolled in the present study.

According to Khormaei et al. Study, if the first type error is 5% and the study power is 90%, the mean score of the first group is 12 and the second group is 10, with a standard deviation of 3 need 41 persons in both groups (82 persons in total). On the other hand, the sample size is increased to 45 persons in each group in order to counter the probable loss of 10% (19).

It should be noted that applying the above equation is equivalent to using the following formula:

$$n=\frac{(\sigma_{1}^{2}+\sigma_{2}^{2}){\left(Z_{1-\frac{\alpha }{2}}+z_{1-\beta }\right)}^{2}}{{\left({µ}_{1}-{µ}_{2}\right)}^{2}}$$

**Fig 1:**
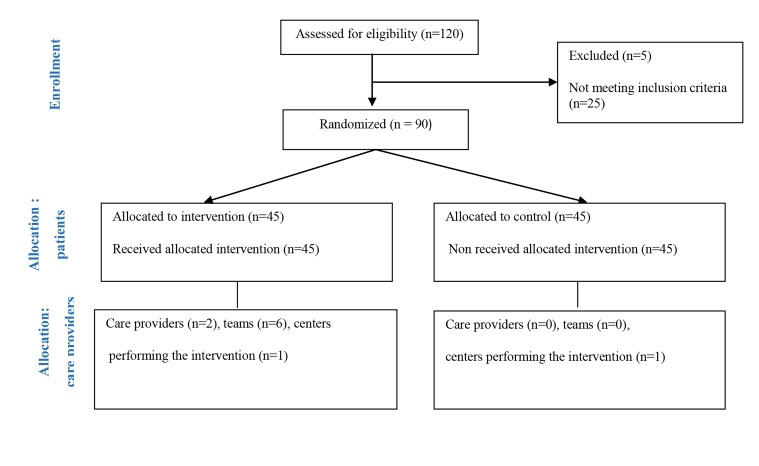
Modified CONSORT flow diagram for individual randomized controlled trials of nonpharmacologic treatments.

After enrollment, the women were divided into intervention and control groups by block randomization, and group counseling was delivered to the intervention group. We constructed 10 blocks of 4 and one block of 5 (45 women), and randomly assigned the participants to the two study groups by assigning the next block of participants according to the specified sequence (Fig .1).

Before starting the study, the aim of the study was explained and verbal and written informed consent was obtained from the women. First, the 45-member intervention group was divided into three 8-member groups and three 7-member groups to increase the efficiency of group counseling sessions. After that, eight 90-minute group counseling sessions were held twice a week (the IVF process can last for 4 to 6 weeks) using mindfulness training packages. Counseling axes included auto-guidance, confrontation with obstacles, breathing with mindfulness, staying in the moment, the untruthfulness of thoughts, and how to take optimal care of oneself (Table 1). These counseling sessions were organized by a researcher trained by a senior researcher with a Ph.D. in clinical psychology, overseen by the professor of psychology in the research team. At the end of each session, an educational note and a CD related to that session were given to participants. During the counseling sessions, participants were divided into small class groups to interact with group members, and state and explain their problems. We used R software (version 3.5.2), a free and open source software for the statistical analysis.

The participants in the intervention group were asked to practice conscious yoga exercises at home and present the principles of counseling, goals, and exercises of the previous session at the beginning of each session. Moreover, in order to resolve possible ambiguities, women in the intervention group were asked to do all exercises in class with the researcher. This resulted in more repetition and training, and helped the creation of a new mindset. During the counseling sessions, we tried to fully explain the meaning and concept of mindfulness through daily routine examples, stories, and conscious yoga exercises. This method was also employed in the infertility center while the women were undergoing their IVF treatment. The control group received routie programs of infertility center and did not recive any intervention. Due to ethical considerations at the end of the study the educational pamphlets and the CD were administered to the control group. Pre-test assessments were conducted on the 90 randomised participants prior to commencing IVF treatments, meaning all members of both groups completed the demographic information and Beck depression inventories. After the intervention the post-test was performed using the BDI-II 3-7 days before the embryo transfer stage. At this point depression is at its minimum and the effect of the intervention on intervention group can be determined better. The counseling sessions are shown in Table 1.

**Table 1:** Mindfulness training taken from Crane R. Mindfulness-based cognitive therapy (20)


Session	Goals	Practical exercise per session	Each session’s program	Homework

1	Automated guidance	Eating a raisin with mindfulness meditation on body checking	Making a group, presenting the moral code of the method and group boundary, introducing participants, providing explanations about infertility and the resulting depression and the necessity of mindfulness training, explaining the automated guidance system	1. Concentration on body checking for 45 min
				2. Attention to daily routines such as daily showers
				3. Eating a meal once a week with mindfulness
2	Facing obstacles	Body checking meditation, 10-minute breathing with meditation and mindfulness	Thinking about practices and the exact feeling of each	1. Reviewing the previous session
				2. 4-minute body checking meditation
				3. 10-minute breathing with mindfulness
				4. Focusing on continuous activity to experience a pleasant day or event
3	Breathing with mindfulness	Conscious breathing and stretching practice, breathing and stretching with mindfulness	3-minute breathing, Identifying and recording pleasant experiences or unpleasant ones to be studied in the fourth session	1. Reviewing the previous session
				2. Continuity and breathing exercises on days 1, 3 and 5 of a week
				3. Practicing movements consciously on days 2, 4 and 6 in a week
				4. Daily recording of pleasant experiences
				5. Three minutes of breathing over three periods of time
4	Staying in the moment	5-Minute seeing or listening with mindfulness 3-minute breathing space, and walking with mindfulness	Discovering unpleasant experiences, detecting and defining depression problems or alternate group focus.	1. Revewing the previous session
				2. Creating relaxation and meditation
				3. 3-minute normal breathing (3 times a day)
				4. 3-minute patterning breathing ( as a meditative strategy while experiencing unpleasant feelings)
5	Acceptance and authorization of presence	Awareness of breathing and body, emphasizing the perception of how to react to thoughts, feelings and body sensations. 3-minute breathing	Reading Guest House poems by Rumi's works and identifying them in the group, practicing the discovery of reactions to normal patterns and the application of the potential talents of mindfulness skills to facilitate the response to the present-day experiences.	1. Reviewing previous session’s assignments.
				2. Thinking in sitting position.
				3. 3-minute normal breathing (three times a day)
				4. Four minutes of patterned breathing (as a meditative strategy in the experience of unpleasant feelings)
				5. Reopening (body doors) and entering the outside realm of the body (in the body)
6	Thoughts do not have a real origin	Meditation sessions, awareness of breathing and body, highlighting the patient's problems during exercise and detecting their effects on the body and mind.	Training in changing behaviors, thoughts, and attitudes, start the development of personal rehabilitation and activity plans, and preparing the participants for the end of the course	1. Reviewing the previous session’s assignments
				2. 40 minutes of daily practice, with different combinations of the three main exercises
				3. Exploring the use of short-term exercises
				4. 3-minute normal breathing (three times a day)
				5. 3-minute patterned breathing (as a meditative strategy when experiencing unpleasant feelings)
				6. Reflection and work on the plan to prevent personal recurrence
7	How can we look after ourselves?	-Meditation sessions- Awareness of breathing, organs, sounds, thoughts, and emotions.-3 minutes of breathing- Highlighting a problem during exercise and detecting its effect on the body and mind.	Discovering the relationship between activity and mood, a general list of daily activities and considerations (emotional drainage) that empowers the body, exploring ways to increase activity (useful), recognizing relapses and activities that cause recurrence.	1. Reviewing previous session’s assignments
				2. The breathing space in accordance with the routine as a coping strategy
				3. Discovering a wa to do dexterous work after practice
				4. Developing an early warning system for recurrence detection
				5. Developing a practical plan that can be used in depressed moods
8	How to use these factors in future decision making	End course body checking meditation	Reviewing early warning system and practical plans (to use in high-risk relapsing time), reviewing all previous sessions, discussing the way of preserving motor power, developed in formal and informal exercises. End of course and acknowledgments.	1. Reviewing previous session’s assignments
				2. Making questions to answer the personal reflections during the day
				3. Doing homework(Mindfulness exercise with booklet study) and practice at home alone (21).


### Data analysis

The Kolmogorov-Smirnov test was used to confirm the normal distribution of all the variables. Data were analyzed includes independent t test and using IBM SPSS V.21, (http://www.meta-analysis.com), to provide descriptive statistics, such as mean and standard deviation, for the quantitative data. Independent tests and Chi-square tests were employed to compare the variables before and after the intervention; paired t tests were employed to compare variations between the groups. The significance level was assumed to be P<0.050.

### Ethical considerations

This study code IR.UMSHA.REC.1395.336 was approved by the Ethics Committee and Research Council of Hamedan University of Medical Sciences. For ethical considerations, at the end of the study, educational notes and CDs were given to the control group.

## Results

In the present study, 90 women meeting the inclusion criteria were divided into two groups of intervention (45 women) and control (45 women); and the effect of mindfulness-based group counseling on depression in infertile women undergoing IVF treatment was evaluated. The mean age of the infertile women in the intervention and control groups was 30.28 ± 5.39 and 29.64 ± 4.71 years, respectively and the mean age of their partners was 34.82 ± 4.97 and 34.37 ± 5.39 years, respectively. Mean marriage duration in the intervention and control group was 8.28 and 8.16 years, and the mean infertile period was 5.26 and 4.39 years, respectively. The majority of infertile women in the intervention (84.4%) and control (71.1%) group were unemployed and most of their partners were employed, 97.8% in the intervention group and 95.6% in the control group. The majority of infertile women in the intervention (57.8%) and control group (57.8%) had a high school diploma. Others had a license and master’s degree; intervention group (40.0-2.2%) and control group (35.6-6.7%) (P=0.08). Most of their partners, 66.7% in the intervention group and 44.4% in the control group, had high school diploma. Others had a license and master’s degree; intervention group (24.4-8.9%) and control group (33.3- 22.2) (P=0.63). Most of the patients in the intervention (86.7%) and control group (62.2%) had health insurance, although most of the treatment costs in both groups were not paid by their health insurance [intervention group (73.3%) and control group (84.4%)]. The frequency of IVF was divided into five categories (0-1, 2, 3, 4 or 5 times): the majority of women in the intervention group had used 0 and 1 times (37.8%) and the majority of subjects in the control group had used 0 times (35.6%) of the IVF treatment. The mean number of previous IVF treatments in the intervention and control groups was 1.11 and 1.24 respectively (Table 2). Mean depressive symptoms scores in the intervention and control group before and after the intervention (the intervention in mindfulness counseling in the intervention group) were 20.77, 10.82, and 17.95, 21.33, respectively (Table 3). Before the intervention the mean depression score was lower in the control group than in the intervention group (P=0.046). As seen in Table 3, there is a significant relationship between before and after intervention in the intervention group (P<0.001), meaning after intervention, the mean depression was significantly reduced. After the intervention the mean depression score in the intervention group was reduced by 48% (P<0.001). In contrast, the mean depression score in the control group had increased by 19% (P<0.001), so that the depression score among women in the intervention group after the intervention was less than half that in the control group (P<0.001). The heterogeity and bias in base line data were solved by using ANCOVA Test.

**Table 2:** Comparison of the mean and standard deviation of certain demographic characteristics (age of men and women, male income, duration of marriage and duration of infertility) in the two groups


Group	Intervention group	Control group	P value

Infertile women’s age (Y)	30.28 ± 4.41	29.64 ± 4.71	0.500
Partners’ age (Y)	34.82 ± 4.97	34.37 ± 5.39	0.680
Partner’s income (Toman)	10681707 ± 2215337.3	1617777 ± 669222.3	0.850
Marriage duration (Y)	8.28 ± 3.45	8.16 ± 4.12	0.870
Infertility duration (Y)	5.26 ± 3.20	4.93 ± 3.38	0.620


Data are presented as mean ± SD.

**Table 3:** Comparison of average depression scores in infertile women before and after intervention in the experimental and control groups


Group	Depression(Before)	Depression(After)	P value for test of difference

Experimental	20.77 ± 6.35	10.82 ± 7.16	<0.001
Control	17.95 ± 6.85	21.33 ± 6.48	<0.001
P value for test of difference	0.0460	<0.001	


Data are presented as mean ± SD.

## Discussion

The aim of the present study was to evaluate the effect of mindfulness-based group counseling on depression in infertile women undergoing IVF treatment. Our results showed that mindfulness-based group counseling reduced depression scores in infertile women. This findings is in line with Hoveyda et al. (21) who measured the effect of stress reduction-based mindfulness and conscious yoga on anxiety, depression, and stress in infertile women, and observed a significant reduction in depression from before to after the intervention in the intervention group. In the present study, 8 X 90-minute sessions of cognitive therapy-based mindfulness counseling were held, while in the mentioned study, there were 8 X 120-minute sessions. However, the content of mindfulness sessions was the same in both studies.

Galhardo et al. (22) studied the effectiveness of mindfulness programs in infertility, and showed a reduction in depression symptoms after the intervention in the intervention group in line with the current study. In addition, their study, like ours, showed an increase in depression scores in the control group after the intervention. Hoveyda et al. (21), in contrast, found no significant difference in depression scores before and after the intervention in the control group. In the present study, there was significant difference between the intervention and control groups regarding the symptoms of depression prior to the intervention. Mentioned study demonstrated that there was no significant difference concerning depression between the intervention and control groups before and after the intervention, which is against of the current results. The study of Galhardo et al. (22) consisted of 55 infertile women in the intervention group and 37 in the control group. The content of this study is similar to that of the present study, including body checking meditation, 3-minute body space, thought and sound meditation, and staying in the present. However, in the study, 10 X 120-minute counseling sessions were held.

Panahi and Faramarzi (17) found a significant improvement in depression symptoms in premenstrual women of the intervention group using mindfulness-based cognitive treatment, compared with the control. Also in findings similar to ours, they found mindfulness-based cognitive treatment to produce a significant improvement in depression symptoms in premenstrual women, P=0.007 compared to control women. Strege et al. (23) showed that depression scores of pregnant women in the intervention group (Positive Affect and Social Anxiety Symptoms) were considerably less than in the control group. We conclude that a counseling approach can play a major role in the reduction of mental disorders such as depression (24) and suggest that it should be included as routine during IVF treatment in infertile women.

One of the limitations of this study was the inadequate completion of the questionnaires (due to their anxiety) by the study sample. In an attempt minimize the error rate in this case, the investigators talked to the participants in the study to resolve this problem and inspire confidence that information would remain confidential. Finally, it was explained to infertile women that reducing anxiety may have the effect of speeding up their pregnancy. Also, due to the length of the counseling sessions (8 sessions), some of the women in the study were not able to attend all the scheduled sessions. To minimize this problem, meeting times were adjusted based on the participants’ suggested time.

## Conclusion

The findings of the present study point to the effectiveness of mindfulness-based cognitive group therapy on depression in infertile women undergoing IVF treatment. Mindfulness counseling reduced depression in the intervention group. In the control group, where no intervention was performed, the depression score increased. As mindfulness-based cognitive group therapy results in a significant decrease in depression symptoms in infertile women under IVF treatment, it is suggested that it should be available to all depressed women undergoing IVF treatment.
